# Large Intestine Model Editor (LIME): Design and pilot evaluation of an editor for parameterized large intestine 3D models

**DOI:** 10.1007/s11517-026-03640-x

**Published:** 2026-08-07

**Authors:** Adrián Lugilde-López, Fernando-Ariel Mikic-Fonte, Manuel Caeiro-Rodríguez, Martín Llamas-Nistal, Antonio Rodríguez-D’Jesús

**Affiliations:** 1 AtlanTTic Research Center, GIST Research Group, University of Vigo, Vigo, Spain; 2 School of Telecommunication Engineering, University of Vigo, Rúa Maxwell, S/N, Vigo, Pontevedra 36310 Spain; 3 University Hospital Complex of Pontevedra, Av. Montecelo, Pontevedra, Pontevedra 36161 Spain; 4 IDARA Research Group, IISGS, Pontevedra, Spain

**Keywords:** 3D modeling, Colonoscopy, Computer graphics, Pilot evaluation, Virtual reality

## Abstract

As virtual training environments for colonoscopy become more prevalent, addressing their limitations such as the scarcity of simulation scenarios and the lack of open, shareable models becomes essential. This paper introduces an editing tool developed to mitigate these issues by enabling non-technical users, such as surgeons, to create varied 3D models of the large intestine. The software employs a parametrized approach using a Bezier spline with control nodes, presets, blendshapes, and specific meshes to enable the generation of a wide range of large intestine configurations, sizes, and pathology conditions. The paper also includes the results of a pilot evaluation focused on the editor utility, model fidelity, and usability, involving 10 colonoscopy practitioners who completed a modeling task and provided feedback via a questionnaire. Results indicate a positive reception, highlighting the realism of the models, user-friendliness, and overall utility. This solution not only addresses key limitations in colonoscopy training, but also lays the foundation for future advancements in colonoscopy simulation technology.

**Graphical abstract:**

**Figure:**
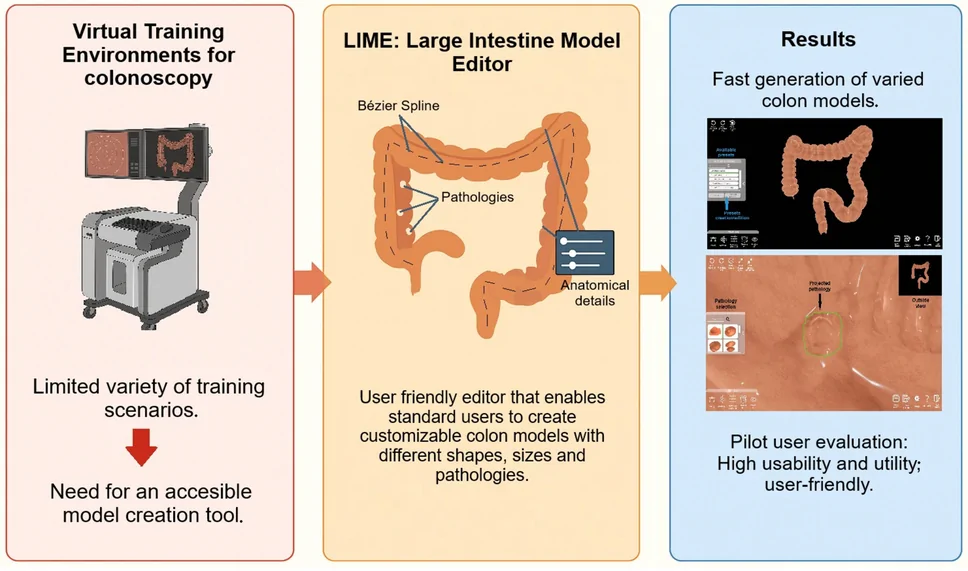

## Introduction

Virtual Training Environments (VTEs) have gained popularity in recent years due to their ability to replicate traditional training experiences in simulated scenarios [1, 2]. Designed for specific fields, VTEs provide simulated scenarios that replicate real-world environments where repetitive training is not possible due to high costs or potential risks. VTEs are being used to assist students in learning complex concepts, particularly in the scientific field, due to their technical difficulty, the need for abstract thought, or the intangibility of these concepts. A key advantage of VTEs is that failure carries no real-world consequences, allowing trainees to practice freely without risk. This fosters skill development by helping trainees recognize potential hazards, correct errors, and recover from mistakes in a controlled setting. In addition, VTEs can offer learner-centered experiences that can be tailored to individual abilities, keeping trainees within their Zone of Proximal Development, known as the gap between what learners can do independently and what they can do with guidance or collaboration with more capable peers [3]. By complementing traditional training methods and reducing reliance on costly equipment, VTEs enhance accessibility, improve learning outcomes, and expand training opportunities.

The adoption of VTEs in the medical field is relatively recent compared to their long history in other training programs [4]. Healthcare professionals have included many forms of simulators in their training programs to improve student education and patient safety. Recent studies have reported the potential benefits [5–7]: risk-free learning environment, enhanced skill development, increased accessibility, cost-effectiveness, stress reduction and confidence building, etc.

In this paper, we focus on VTE solutions to support the training of colonoscopies, also known as lower gastrointestinal endoscopies (LGIE) [8]. Colonoscopy is an invasive procedure involving the use of a flexible endoscope to examine the colon. Since its introduction in 1969, this method has become the gold standard for colon screening programs. Innovative colonoscopy simulators have recently been developed to take advantage of the benefits of VTEs and support the training of colonoscopy practitioners. The efficacy of these simulators as educational tools has already been acknowledged in the medical literature [9–11].

A main issue regarding existing colonoscopy VTEs is the scarcity of training scenarios. Their ability to repeat the same scenario to analyze mistakes and learn from them is also crucial for novices, as demonstrated by Issenberg et al. [12]. However, novices should be exposed to a wide variety of cases in order to acquire colonoscopy skills. Every patient is unique, as are their pathological conditions. Therefore, colonoscopy VTEs should be able to provide a variety of training scenarios and exercises for every aspect of the procedure, such as variations in anatomy, appearance, pathologies, and physical characteristics.

**Fig. 1:**
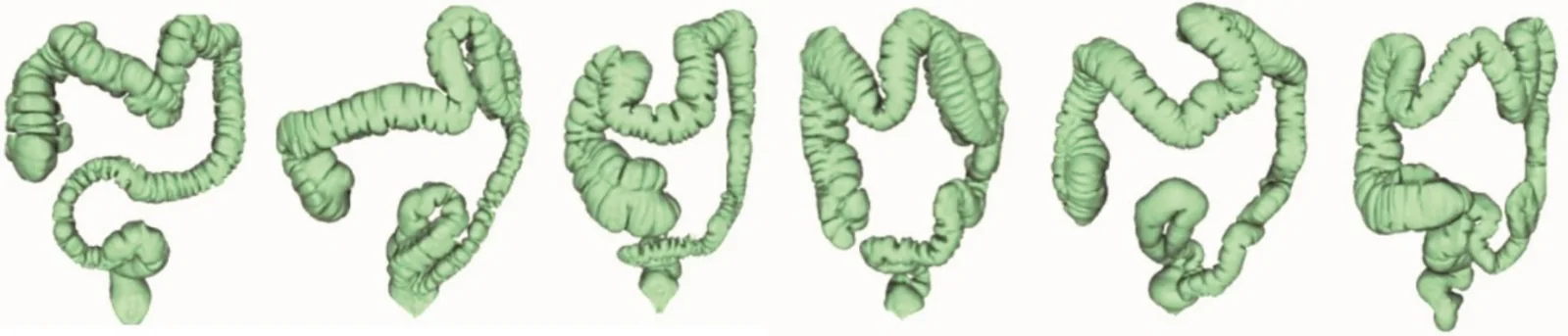
Large intestine models reconstructed from real patient cases (adapted from [16])

The limited number of training scenarios in simulators is mainly related to the complexity of obtaining quality large intestine models (for instance, commercial VTEs such as EndoVR [13], GI Mentor [14], and EndoSim [15] offer the trainees a small set of exercises reproducing some typical problems or situations representative of the colonoscopy practice). The large intestine has a complex shape, and in some patient cases, the shape can be quite peculiar (Fig. 1). Therefore, the manual modeling of these large bowel models with 3D editing software requires significant time and expertise, making production or modification challenging for non-technical users and leading to high costs.

To address these limitations, this paper introduces the Large Intestine Model Editor (LIME), a key component of the LoGiViT (Lower Gastrointestinal Virtual Trainer) system [17]. LIME aims to aid the colonoscopy simulation field through the following key features:

- Usability for non-technical users. LIME enables users without technical expertise in visualization technologies (e.g., surgeons) to create high-fidelity large intestine models.
- Unlimited models creation. In contrast to existing colonoscopy VTEs which are limited to a fixed set of models, LIME enables the creation of an unlimited number of customizable models with specific bowel tracts, shapes, sizes and pathologies.
- Independence from medical imaging. Unlike conventional pipelines, LIME generates fully editable 3D colon models parametrically, removing the need for Computed Tomography (CT) or Magnetic Resonance Imaging (MRI) data, along with its associated costs and privacy issues.
- Open architecture. To support future contributions from third parties, LIME and LoGiViT are developed within the Unity framework, a well-known 3D engine [18], with strong support for third-party extensions.

The remaining sections of this paper are structured as follows. The next section details a brief state of the art of colonoscopy simulators and the advances in 3D organ modeling. Section 3 describes the implementation of the LIME and its features. Section 4 explains the LIME pilot evaluation and its results. Section 5 analyzes these results, and Section 6 closes the paper with some conclusions.

## Related work

Before introducing LIME, we review the existing solutions in the medical field to both assess the state of the art and highlight the gap that LIME fills. Section 2.1 examines existing virtual reality and computer-based colonoscopy simulators, focusing on the underlying modeling strategies, visual fidelity, and the presence of model editing tools. Section 2.2 then surveys the state-of-the-art pipelines for creating 3D organ models, highlighting the ongoing reliance on expert manual intervention and the lack of end-to-end workflows accessible for non-technical users. These overviews show the novelty of our work, and exposes the critical gap that LIME is designed to fill: a user-driven colon model editor with a streamlined, user-friendly parameterized mesh generation.

### Virtual colonoscopy simulators

The current generation of colonoscopy training simulators is dominated by commercial VR and computer-based platforms that recreate endoscope navigation within pre-built colon models. Key systems are:

- EndoVR [13]: A simulation system that combines force-feedback with an integrated virtual patient monitor. The software simulates tissue deformation and loop formation via physics-based algorithms, and it can display vital sign changes such as heart rate spikes if excessive force is applied. Instructors can toggle settings such as bleeding or mucosal tears to adjust the difficulty of the case, yet the scenario is fixed and does not provide support for user defined models.
- GI Mentor [14]: A compact high-fidelity simulator that combines a real endoscope with physics-based 3D rendering of colon anatomy. Trainees experience realistic looping, friction, and collision feedback while practicing different colonoscopy tasks (vendor-locked, available as purchasable modules). Instructors can adjust difficulty parameters such as tortuosity and patient body type, but it offers no means to import models or create new ones. Extensive validation studies confirm its validity, but its closed library limits scenario diversity.
- EndosSim [15]: A portable simulator featuring a compact haptic unit into which a real endoscope is inserted. The system reproduces realistic insufflation dynamics, scope recoil, and high-fidelity textures, with modular exercises configurable by instructors. They can choose difficulty levels by altering the loop formations and disease prevalence, done by changing between available models, but user importing or editing of the underlying meshes is not available.

Along with these commercial simulators, there are a few promising research prototypes that explore new solutions:

- Mikoto [19]: A hybrid physical-computerized simulator in which trainees insert a real endoscope into a colon silicon model. Embedded pressure and motion sensors together with actuators and positional shifts work with an artificial intelligence driven software to manage forces, loop formation, and patient discomfort. Instructors can adjust difficulty by swapping parts of the silicon model with varying tortuosity, but there is no software-based model editing.
- ViGaTu [20]: An immersive virtual reality system that uses a head-mounted display and a motion-tracked endoscope handle. It simulates not only the colonoscopy navigation, but also the complete clinical workflow including patient consent, sedation managements, vital sign monitoring, and team communication. Instructors can select from built-in scripted scenarios (e.g., colonoscopy with multiple small polyps), but the system lacks any user tools for creating or modifying the colon models.

Despite remarkable advances in haptic realism, visual fidelity, and the integration of emerging technologies, such as AI systems for the automatic detection and characterization of malignant pathologies [21], none of these simulators or technologies offer a user-friendly solution for creating or customizing colon models. This limits the diversity of training scenarios and makes it difficult to rapidly generate patient-specific or new colon models.

### 3D organ modeling

For the past two decades, the dominant workflow for creating patient-specific 3D organ models has consisted of computed tomography (CT) or magnetic resonance image (MRI) segmentation followed by surface reconstruction and extensive mesh repair [22–24]. These 3D models have proven to be useful in clinical settings, such as organ 3D printing in preclinical settings [24] or in aiding the localization of complex pathologies for surgical planning [25]. In this model generation workflow, experts manually annotate hundreds of these 2D images for each patient case on dedicated software, and the resultant binary mask is then converted into a polygonal surface (mesh). Then, a modeling technician must repair the mesh by closing holes, removing self-intersections, adding texture coordinates, and several additional steps before achieving a valid organ model. This process requires specialized skills, is labor intensive, and time consuming, particularly for organs with complex anatomies such as the colon. Academic and industrial groups have tried to speed up several tasks of this workflow in an effort to streamline and simplify it through several key approaches:

- Segmentation automation. Early methods combined thresholding, region growing, and edge detection to reduce manual effort, with platforms like Biomedisa [26] enabling interpolation of 2D image sets from sparse labels. More recently, the application of deep learning has become the main focus. Convolutional neural networks (CNNs), such as U-Net and nnU-Net, could be used to generate high-quality segmentation masks for single or multiple organs [27, 28]. The most recent efforts even explore natural language and multimodal interfaces to guide segmentation [29, 30]. However, due to the recent emergence of these technologies, most of these methods remain in prototype stages and have not seen widespread adoption, while remaining fundamentally dependent on the availability and quality of source imaging data.
- Surface reconstruction and refinement. Once segmented, the data must be converted into a polygonal mesh. Techniques such as skeletonization have been used to extract centerlines for simplifying the meshing of tubular structures such as the colon [31] or vascular vessels [32]. Other hybrid techniques combined CNN outputs with region based smoothing or morphological filters to correct artifacts [33]. Recently, more advanced deep learning networks were proposed to automatically detect and repair topological defects in generated structures, reducing the need for manual mesh fixing [34]. Despite these advances, model preparation (e.g., smoothing, remeshing, texture mapping) remains a manual step.
- Direct reconstruction methods. To bypass the segmentation bottleneck entirely, some researchers have used statistical shape models (SSMs) and deep generative models. For instance, SSMs were successfully applied to reconstruct complex bone structures from partial surface data [35]. Other works leverage neural representations like signed distance fields or infer manifold meshes [36]. While promising, these methods typically infer shapes from learned statistics rather than allowing user-directed creation.

Despite these promising results, three practical issues remain for creating organ models:

- Quality control: Even with deep learning, automatic outputs often produce mislabeled regions, collapsed loops, or stray fragments that require manual inspection and correction before reliable use.
- Model preparation: Even perfectly segmented volumes require smoothing, remeshing, and texture mapping to become assets ready for simulation. No current tool automates this process end-to-end, nor does it work for all the organ types given their anatomical variability.
- Data availability and privacy: All of the above methods depend on access to CT or MRI scans of high quality and artifact-free. This can be difficult to obtain at scale due to imaging costs, along with legal restrictions on patient data privacy.

In conclusion, despite the significant progress, no off-the-shelf tool universally converts the 2D images into an organ model, let alone an editable asset. This is also reflected in the reduced catalogue of organ models available in medical simulators, along with the scarcity of public datasets. Until the organ models can be generated from higher level specifications rather than low level edits, the field will keep facing a model variety bottleneck.

## Large intestine model editor

To fill the gap identified above, we developed LIME, an editor that lets any user create and modify realistic colon models on the fly, without depending on CT or MRI scans, segmentation, or any of the traditional techniques. The main goal of LIME is to enable regular users (colonoscopy practitioners) to create computerized models of the large intestine. In addition, LIME should provide the means to modify the large intestine models in a simple and efficient manner. This is essential to enable the creation of a wide variety of large intestine training models and, consequently, achieve a higher level of training adaptability, a key feature desired for colonoscopy VTEs. More specifically, LIME was designed to meet a number of essential requirements:

- No technical knowledge should be required to use the editor.
- It must be user-friendly, with an adequate learning curve and a short average time required to create/edit any model.
- It should allow the creation of a broad variety of models regarding their size, shape and anatomic details.
- It should provide the means to interact with the elements used in the generation of the bowel model.
- It must provide methods for adding several disorders to the intestine in order to generate functional models for colonoscopy training.
- The underlying data and models should be sharable between users and should have an easily comprehensible format.

Taking these requirements into account, the editor was designed to allow the creation and modification of intestine models, along with standard options to manage the stored models (e.g. import and export) using JSON format (machine and human-readable). New models are created by modifying a default canonical model, offering later the possibility of modifying these stored models through the use of several editing modes, each of them with its specific types of available modifications, addressed in the following sections.

It is important to highlight that we followed an iterative process, where the development and design of LIME were guided by the continuous feedback of a colonoscopy expert. This specialist was involved from the initial design stage to the implementation and testing of the initial prototypes, resulting in a streamlined form of feedback before conducting the pilot evaluation. He offered support not only in defining critical functionalities but also in ensuring that the tool aligned with the colonoscopy training need. This feedback included suggestions for refining the meshes to improve anatomical realism, provided initial assessments of visual quality, and outlined requirements for tools that would enable the addition of pathologies to the models, among others. This approach ensured that LIME was not merely an adaptation of already existing tools, but a refined solution designed to facilitate the development of large intestine models for colonoscopy training.

### Large intestine model generation

**Fig. 2:**
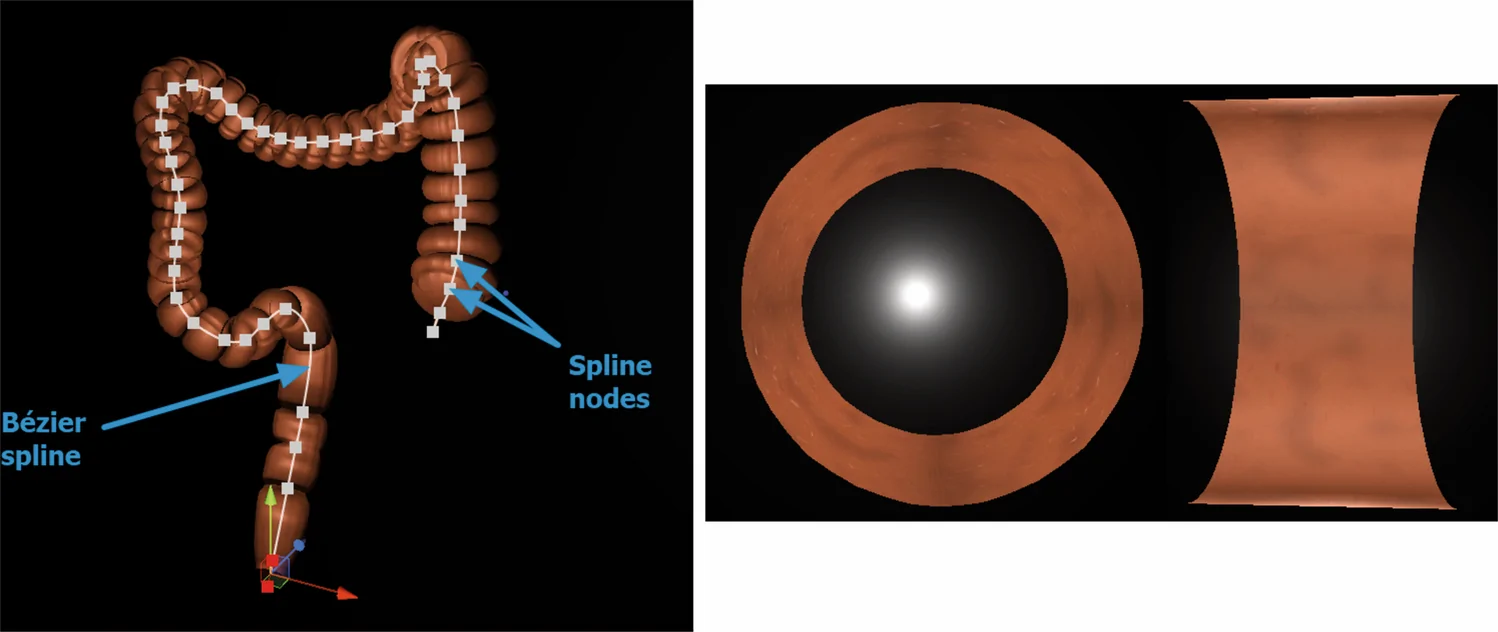
On the left, the generated large intestine model in its canonical shape. On the right, frontal and lateral view of the mesh for the haustra mesh in its default shape

The large intestine virtual model is constructed using a 3D mesh, where vertices are connected by edges to form polygons (triangles) that represent organ’s inner surface. However, instead of modeling the large intestine as a 3D object, LIME represents it as a wrapped path with a defined starting point (rectum) and endpoint (cecum). The core principle of the developed approach involves creating an editable path and wrapping the large intestine elements (e.g., haustra, taenia coli) around it to generate a complete 3D model. For this purpose, a Bézier spline is used as the path, and the organ is discretized into a series of haustra sections, as shown in Fig. 2. Each section, derived from the same haustra mesh, is seamlessly connected to its neighbors in sequence to form a large intestine mesh. Currently, LIME is using high poly meshes with up to 132,480 polygons.

### Editing modes

The editing modes represent the stages into which we divided the process of editing a model of the large intestine. This division not only allowed us to partition the editing process into distinct contexts with their own functions, but it also made the editor more intuitive. LIME offers the following five editing modes: i) tract; ii) presets; iii) anatomical details; iv) pathologies; and v) preview. The first four modes of editing represent the expected visual editing procedures (set a shape, set the details, and place pathologies). The last mode (preview) is always accessible and serves as a support mode for the other four.

#### Tract editing mode

As the first step of the editing procedure, the purpose of tract editing mode is to provide the means to edit the Bézier spline: the core of the model. The editor provides “Move” and “Rotate” options for a set of nodes along the spline. The "Move" option enables changing the 3D position of the spline nodes, thereby deforming the linked meshes to conform to the new spline shape and defining a new bowel tract. Conversely, the "Rotate" option rotates the spline nodes, thereby rotating the linked meshes. A "Joined nodes" option was also included to ease modifications over a set of nodes. When enabled, modifications made to a spline node are partially propagated to its neighboring nodes to ease the editing process. In the settings of the editor, it is possible to change the ratio of the modification that is inherited and its range. In addition, a mechanism was included to limit the maximum displacement of the nodes to avoid unrealistic shapes.

**Fig. 3:**
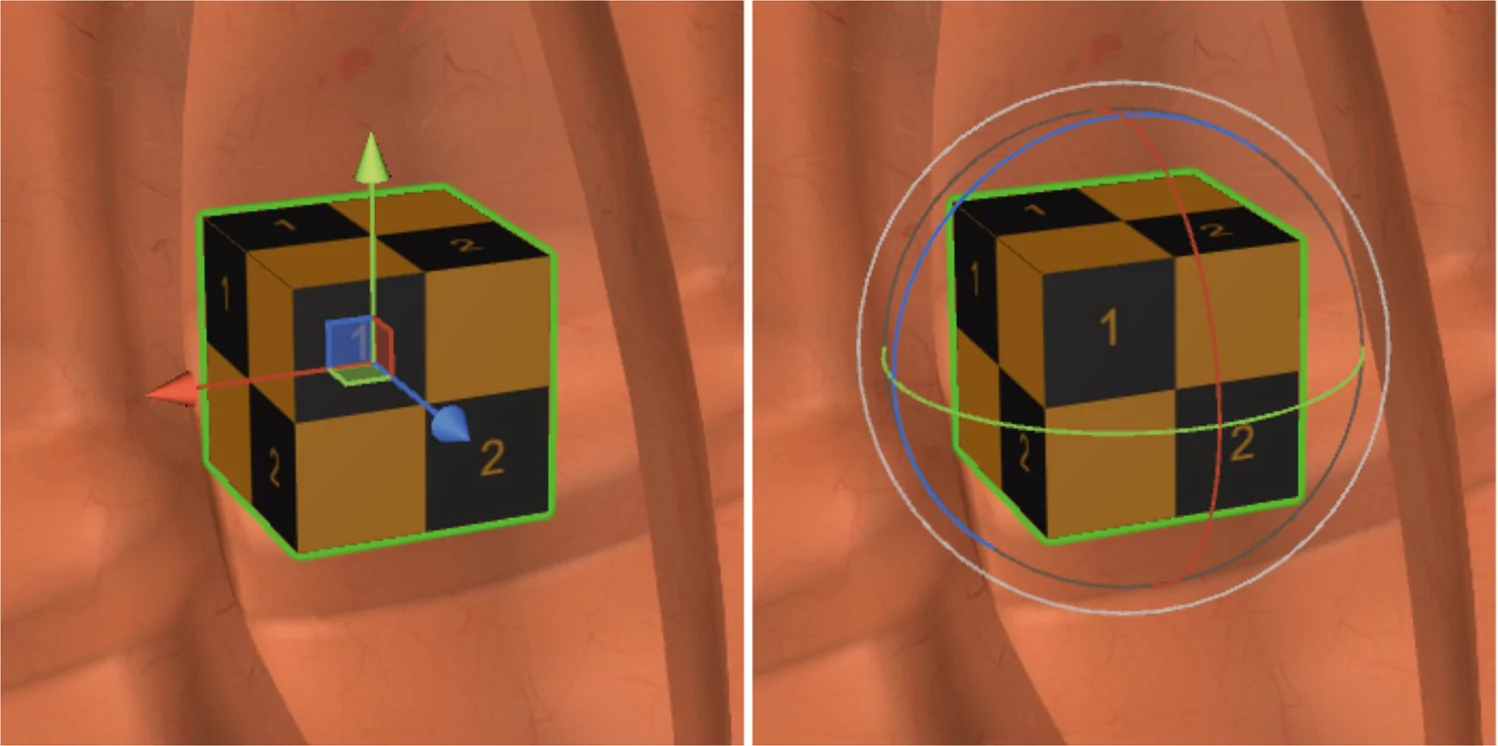
Editor gizmos from left to right: move and rotate

Handlers (cubes, Fig. 3) are created in the 3D space location of the spline anchor nodes to allow the interaction with the spline. On selecting any handler, a transform modification gizmo appears (colored lines, Fig. 3). By manipulating the gizmo (selecting and dragging), the selected handler will be moved or rotated, as will the linked spline nodes, either in one or multiple axes simultaneously.

#### Preset editing mode

The large intestine has a set of anatomical configurations commonly observed in patients (with minor variations), such as Alpha loops and N loops [37, 38]. These configurations could be generated using the previous movement options, but the process could be burdensome. To ease their modeling, LIME includes the preset editing mode, where presets of these anatomical shapes and other variations can be created and saved to be used when needed.

**Fig. 4:**
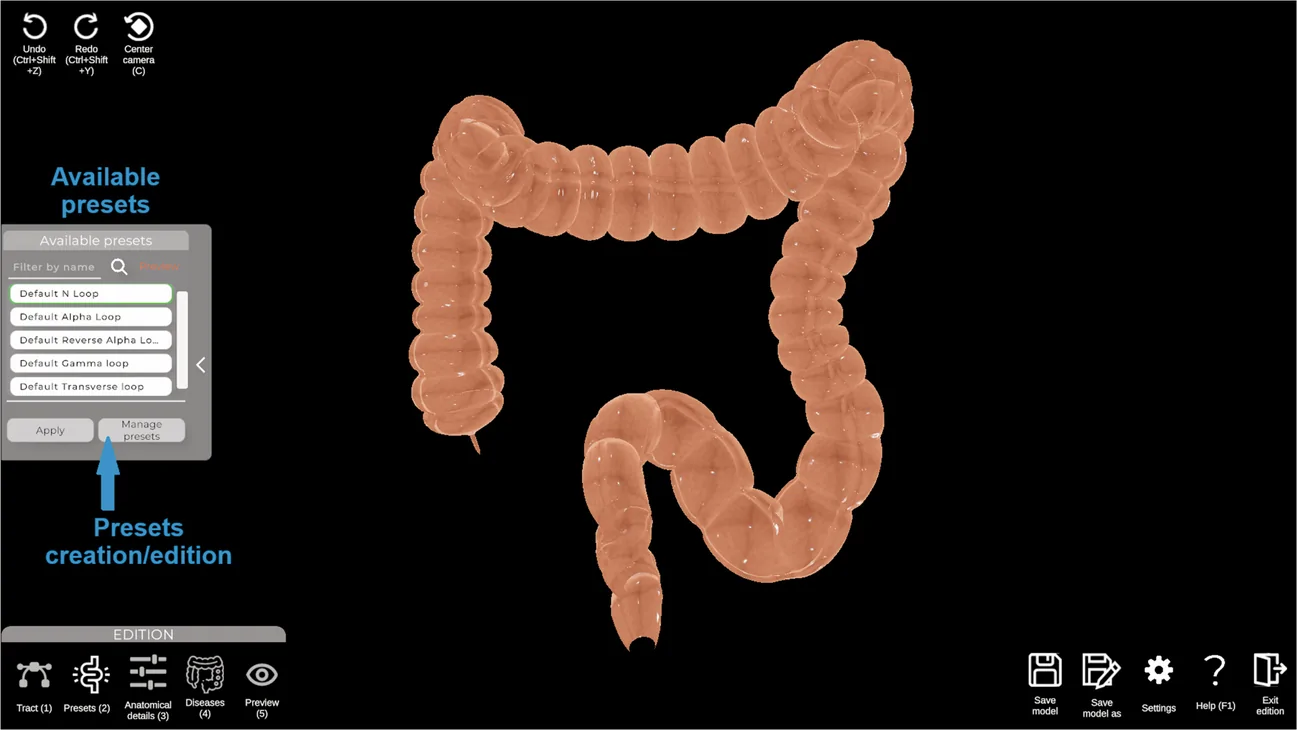
Initial screen of the editor in the preset editing mode

When accessing the preset mode, the available presets are listed and displayed in a panel alongside the management options (Fig. 4). The editor includes several presets by default, such as Alpha loop, Reverse Alpha loop, and N loop. When one of the available presets is selected, it is displayed in the model as a preview. If the preset is not applied, changing the selection or entering a different editing mode will remove it, reverting the model to its state prior to the preview. Editing or creating a preset enables the previously described spline editing features (e.g. move and rotate options, handlers). None of the modifications done to the model during the creation or editing of presets are applied to the final model unless the preset is applied after saving it.

#### Anatomical details editing mode

As stated previously, the large intestine is an organ with complex anatomical structures. In order to reproduce them our haustra mesh derived in submodels (generic, rectum, or cecum), with a wide variety of blendshapes that provide the necessary variations in anatomical details (Table 1). These blendshapes are predefined sets of mesh geometry bound to a default mesh by a strength value. Blendshapes are created based on the morphing technique, deforming a default mesh to obtain multiple predefined shapes and allowing deformations as combinations of several of these blendshapes. Changes to the strength value of the blendshapes determine the degree to which they influence the default mesh. Any intestine model created in the editor has blendshape weights that are set to the values specific to a model (if editing a model) or to default ones (if creating a new model).

**Table 1:** Blendshapes included in the haustra submodels and their function

Submodel type	Name of the blendshape	Function
Shared for generic, rectum, and cecum	Inferior union border	Deforming the mesh borders to represent the folds in the tissue between adjacent haustra
	Superior union border	
	Inferior union border aperture	Deforming the mesh borders to control the aperture between adjacent haustra
	Superior union border aperture	
	Inferior tenia	Deforming the walls of the mesh to change the presence of tenia along the haustra
	Superior tenia	
	Shape	Smoothing the haustra walls to achieve a more rounded, uniform shape
	Union border aperture ease	Controlling the deformation near to the borders to ease the transition to the target aperture
	Global widening	Increasing the width of the haustra mesh
	Global narrowing	Reducing the width of the haustra mesh
	Inferior section width X	Controlling the width along the X-axis of the haustra mesh in its different sections
	Middle section width X	
	Superior section width X	
	Inferior section width Y	Controlling the width along the Y-axis of the haustra mesh in its different sections
	Middle section width Y	
	Superior section width Y	
Exclusive to rectum	Rectum aperture	Deforming the inferior border of the mesh to control the aperture of the rectum
	Rectum canal	Deforming the walls of the haustra mesh to form the rectum canal
	Up and low Houston valves inferior section aperture	Deforming the mesh borders to control the aperture between adjacent haustra to form Houston valves
	Up and low Houston valves superior section aperture	
	Middle Houston valves inferior section aperture	
	Middle Houston valves superior section aperture	
	Up and low Houston valves inferior section shape	Deforming the shape and position of the Houston valve aperture
	Up and low Houston valves superior section shape	
	Middle Houston valves inferior section shape	
	Middle Houston valves superior section shape	
Exclusive to cecum	Appendix shape	Deforming the mesh of the haustra to shape as an appendix
	Appendix folds	Deforming the mesh of the haustra shaped as the appendix to add folds in the tissue
	Cecum aperture	Deforming the mesh borders to control the aperture in the haustra that connects with the one shaped as appendix

**Fig. 5:**
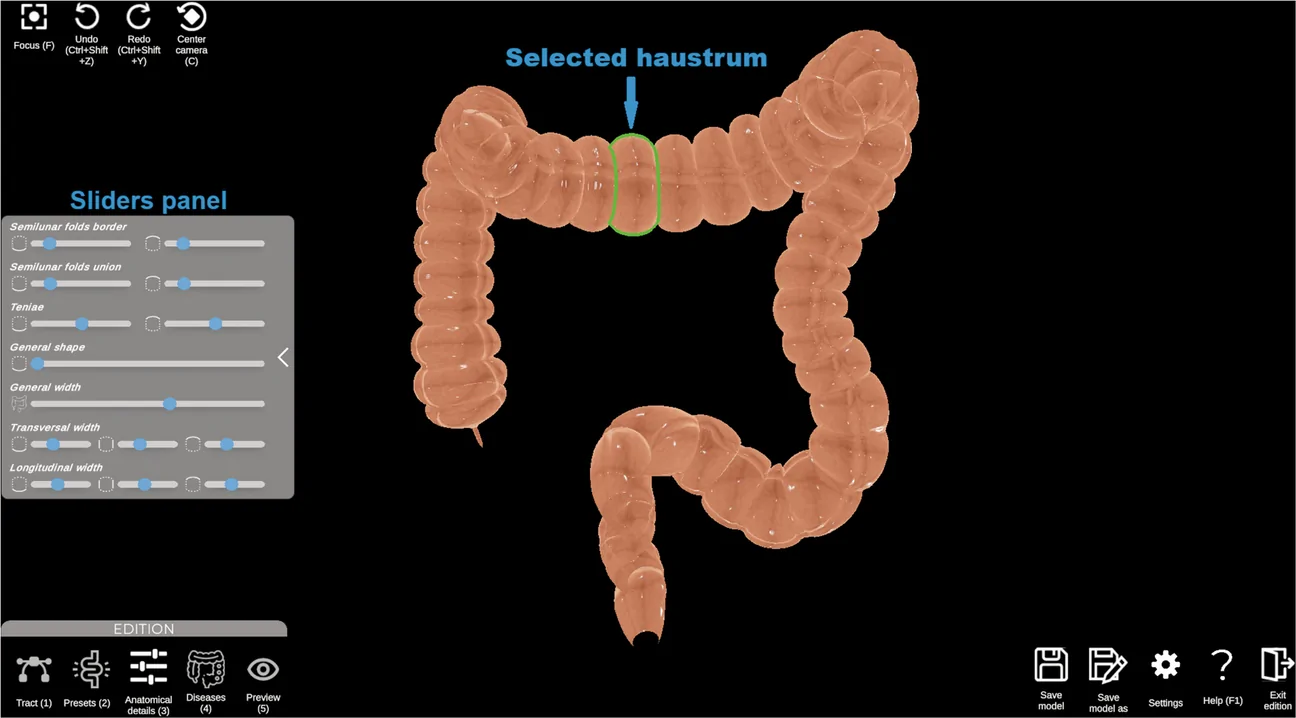
Initial screen of the editor in the anatomical details editing mode

A panel containing a set of sliders is displayed when selecting any submodel of the large intestine model in the anatomical details mode (Fig. 5). Sliders are used to simplify the adjustment of the blendshapes because they employ a value that can be linked to the blendshapes weight. The sliders vary depending on the submodel type. Some sliders are connected one-to-one to the selected submodel blendshapes, while others are connected one-to-many. Furthermore, most sliders affect specific sections of the mesh, like the inferior or superior half or the edges of the apertures. By manipulating the slider handle (selecting and dragging), the blendshape weight is updated, and the associated shape change is updated at runtime.

#### Pathologies editing mode

Multiple types of bowel disorders from a set of predefined ones can be included. To include a disorder, the user is prompted to an inside view of the intestine model, allowing the user to freely navigate along the bowel tract, similar to a colonoscopy view. In this view, the user can select and position any of the available disorders. As the intestinal walls of the model are not smooth, the pathology mesh is deformed to fit the shape of the bowel at the desired location. Once the disorder mesh has been deformed, it is merged with the geometry of the large intestine mesh.

**Fig. 6:**
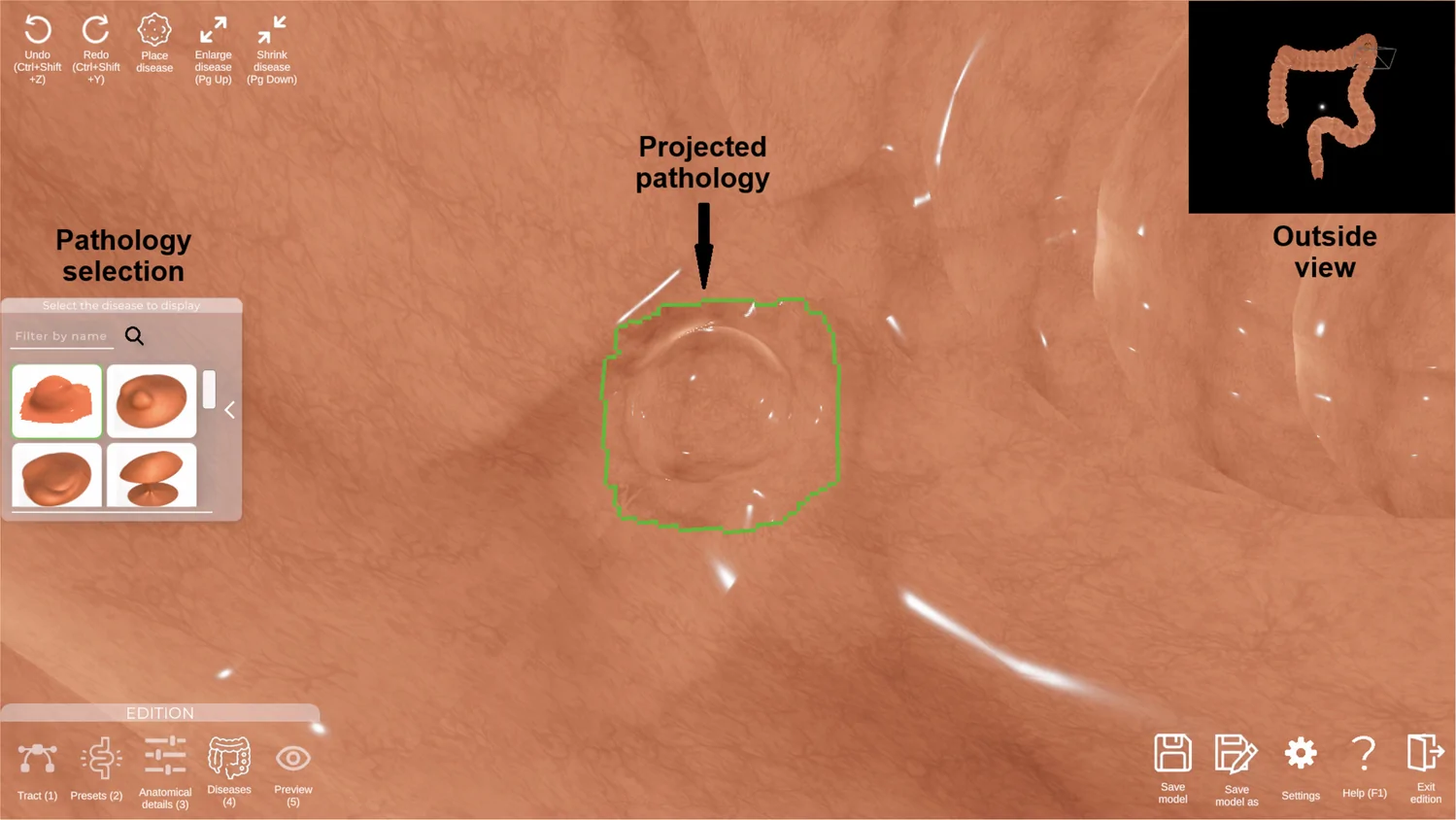
Initial screen of the editor in the pathology mode

Accessing this mode enables free movement using the guided camera navigation option. If a pathology from the displayed menu is chosen, it is projected and wrapped around the intestine model in runtime, following the camera movement. The pathology mode is also used to place the ileocecal valve in the cecum section of the large intestine model, since providing the necessary position variations for this valve with blendshapes alone is not feasible. Figure 6 depicts the user-view in this mode.

**Fig. 7:**
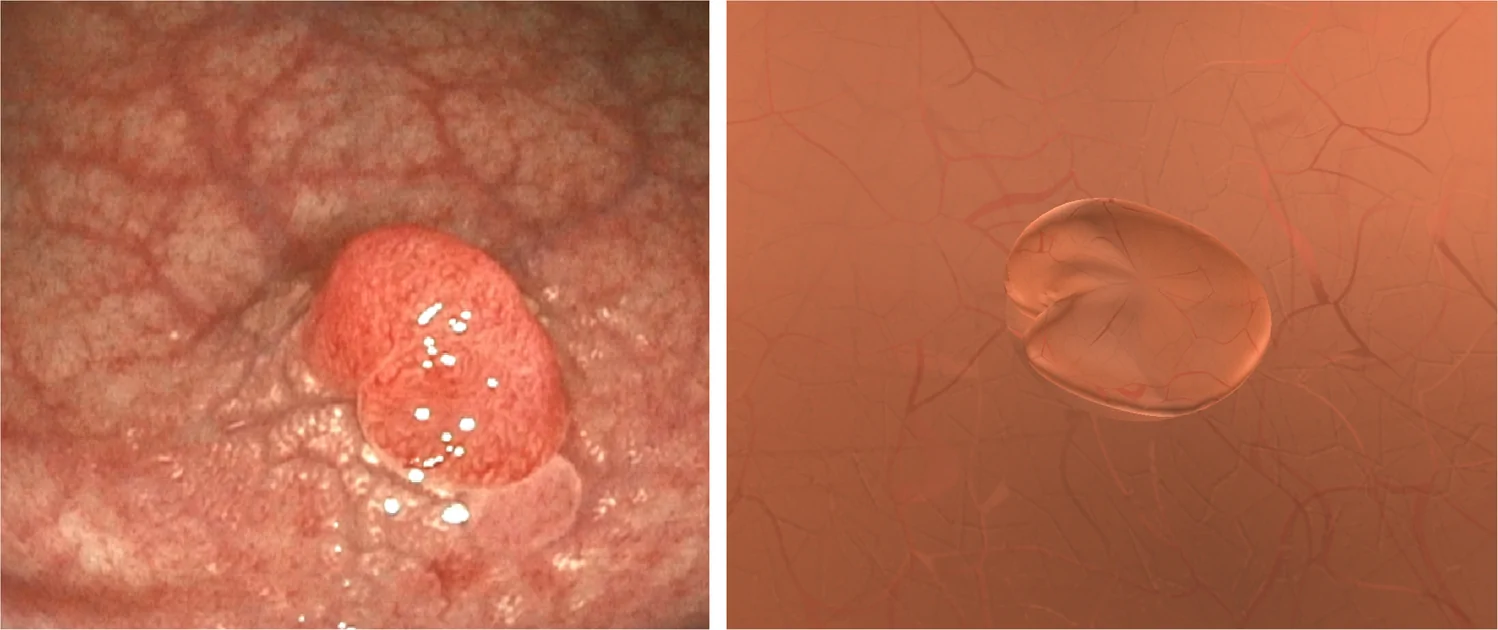
On the left, the source snapshot of a real polyp during a colonoscopy procedure. On the right, the virtual polyp generated and rendered in LIME

To achieve better visual fidelity, the pathology models were designed using real colonoscopy footage as ground truth. In the current version, the editor includes a catalogue of exclusively polyps, categorized by four standard clinical features: size, location, Paris classification, and JNET classification. To ensure the anatomical consistency of the original models is preserved during the placement process, the system uses a deformation threshold. This mechanism ensures that while the pathology blends into the intestinal wall, its morphological features avoid unrealistic distortion, disabling placement when the deformation goes over the threshold. Figure 7 exemplifies this with a side-to-side view of a real polyp snapshot and its virtual counterpart rendered in LIME. Despite not being the focus of the current version of the editor, it can be observed that the editor mimics key visual features while seamlessly blending the pathology edges with the intestinal wall.

#### Preview mode

When this mode is activated, a visualization of the model is displayed without any user interface editing panels or gizmos. The camera can switch between an exterior and an inner view of the model. The inner view resembles the guided navigation view in the pathologies editing mode. From the exterior view, a specific haustrum can be chosen to begin the guided inner navigation from it, or none can be chosen to begin from the rectum.

### Technical performance

To measure the efficiency of LIME, objective performance metrics were gathered using commodity hardware. Specifically, the system used was a laptop equipped with an Intel Core i7-11800H CPU, Nvidia 3070 GPU and 16 GB of RAM. Despite the high polygon count meshes used (up to 132,480 polygons), the system achieved real-time responsiveness, with model deformation and blendshape changes updated at runtime without perceptible latency. On the tested hardware, the editor maintains a consistent frame rate of 60 FPS during interactive editing when using vertical synchronization (VSync). Furthermore, the resulting model data stored remains lightweight, typically under 2500 KB, facilitating easy sharing and storage.

## Lime: Pilot evaluation

With the addition of simulators to the medical field training programs, a major concern is to assess their usability and the user expectations. It is generally accepted that simulators need to undergo a pilot evaluation, as this preliminary feedback is essential for identifying design flaws and optimizing training systems early [39–44]. This ensures that the simulator meets end-user expectations before a comprehensive validity check is conducted [45–48] and as a reference for future application developments. Based on this prior work, we developed and conducted our own pilot evaluation plan to answer the following questions:

- Will the subjects consider the editor useful as a tool for recreating specific patient cases?
- Will it be considered equally useful by novices and experts?
- Will it be considered user-friendly by the subjects?
- Will the subjects favorably value the geometric fidelity of the models?
- Will they assess the editor as a useful tool to achieve higher training adaptability?

These questions aim to evaluate the utility, feature relevance, and usability of the LIME in our simulator. As shown in Section 2, none of the leading endoscope simulators provide users with tools to create or modify colon models, making our editor an unexplored solution. Therefore, this approach based on gathering and examining the opinions of experts and novices, seemed the best option before continuing with further developments. Additionally, we posed three hypotheses from the questions:

- Subjects will consider the LIME a useful tool for the creation of large intestine models.
- Subjects will favorably value the quality of the models.
- Subjects will favorably value the usability of the editor.

Regarding the protocols for the evaluation, we drew our standards from a range of simulation validation guides and studies [39–48]. As a result, the evaluation protocol established was as follows:

- All participants received an explanation of the characteristics and purpose of the pilot evaluation and LIME was presented, with a brief explanation of its functionalities, use instructions, and workflow.
- The exercise to be completed was then introduced. This exercise was a step-by-step guide to test all tools within the editor with the goal of designing their own large intestine model or to replicate a model provided as example. This approach not only allowed users to test the functionalities of the editor, but also to assess the quality of the models in the editor context. Testing in the dynamic simulation context of a simulated colonoscopy procedure could provide additional feedback but would also introduce certain visual restrictions regarding the models. Therefore, evaluating the models directly within the editor allowed users to assess the anatomical correctness and realism of the models without the restrictions of a simulated endoscopy view.
- After completing the exercises, the participants answered a custom-made questionnaire regarding their experience composed of both 5-point Likert scale and short questions. We drew the questions from the simulation validation literature [39–48], adapting them and adding new ones to fit the context of LIME.

## Results

The evaluation was carried out in the premises of the Digestive Care Unit of the Hospital Álvaro Cunqueiro, Vigo, Spain, between April and July of 2023. The commodity PC described in Section 3.3 with generic mouse and keyboard devices was used to execute LIME. Ten colonoscopy practitioners participated in the evaluation and completed the survey. Answers were gathered and analyzed, computing mean and standard deviation (SD) values for the pertaining survey questions.

Subject ages ranged from 25 to 35 years (*n = *3) and 36 to 55 years (*n = *7). Only one subject reported prior experience with computerized colonoscopy simulators (GI Mentor II, from Simbionix Inc.). Most subjects (*n = *8) reported very limited prior experience with other 2D/3D editors (mean 1.3; SD = 0.64), with only two subjects reporting some experience with this type of software. On the other hand, most subjects (*n = *8) reported average experience with mouse and keyboard computer games (mean 2.7; SD = 1.19). Almost all subjects (*n = *9) had performed colonoscopy procedures in the last year. Colonoscopy novices (*n = *2) performed more than 100 and less than 200 colonoscopies, while experts (*n = *7) performed more than 200 (four subjects performed over 500). One subject reported no colonoscopy experience but had participated as an observer in several procedures (less than 25, also considered as novice).

Regarding the provided exercise, subjects reported that additional instructions were not significantly required to complete it (mean 3.4; SD = 0.8). The time spent to finish the exercise was rated as adequate (mean 4.1; SD = 0.54), as was the complexity of the exercise (mean 4.1; SD = 0.7).

**Fig. 8:**
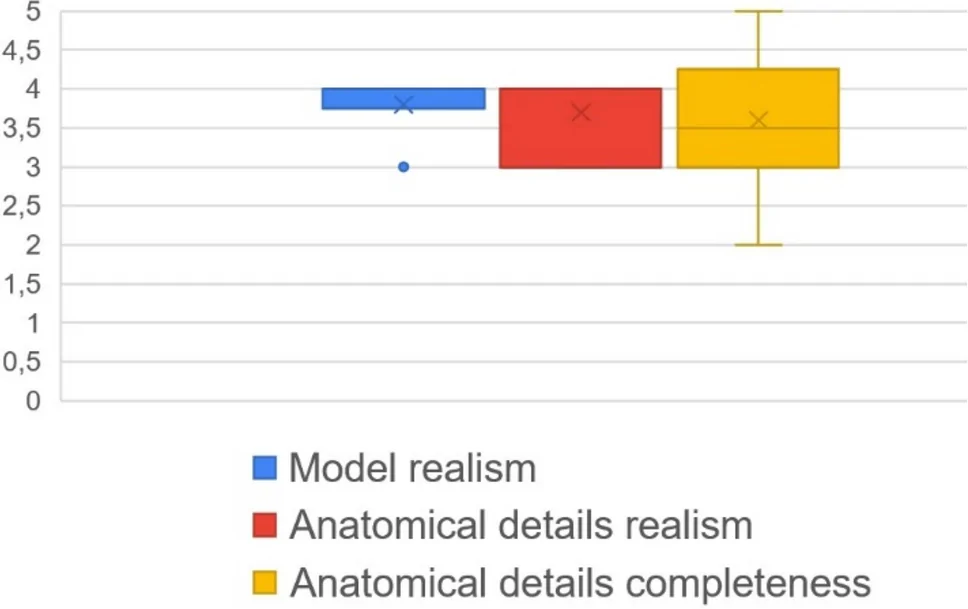
Realism related answers boxplots

In relation to the realism of the editor (Fig. 8), subjects rated the model shape, excluding textures and colors, as realistic (mean 3.8; SD = 0.4). The anatomical details of the model (e.g., tenias, Houston valves, folds) were also rated as realistic (mean 3.7; SD = 0.46). Subjects answered that most of the required anatomical details were present in the models (mean 3.6; SD = 0.91), with only the splenic and hepatic imprints missing.

**Fig. 9:**
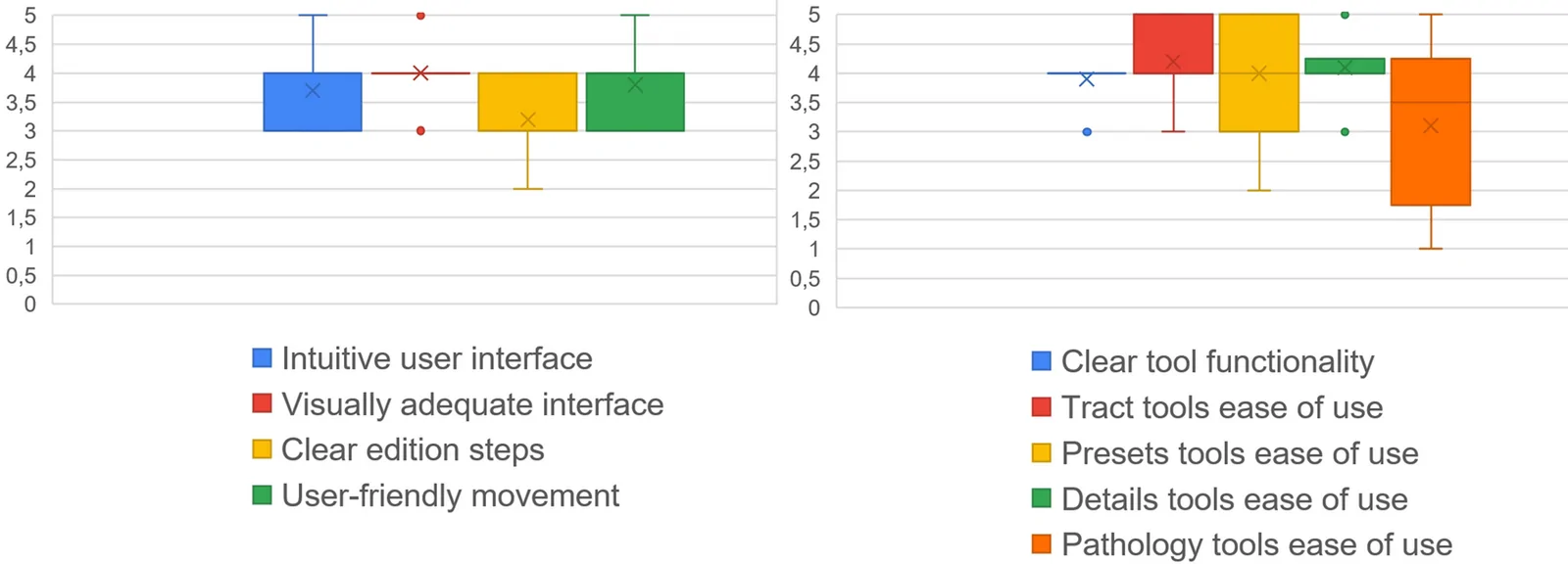
User-friendliness related answers boxplots

Regarding the user-friendliness of the editor (Fig. 9), subjects rated the user interface as intuitive (mean 3.7; SD = 0.64) and visually adequate (mean 4; SD = 0.45). The editing steps were rated as clear to follow (mean 3.2; SD = 0.6), and movement on the editing environment was positively rated (mean 3.8; SD = 0.6). Subjects reported that the editor tools functionality was clear (mean 3.9; SD = 0.3), being all the tools easy to use except the pathology related tools for which opinions were mixed (mean 3.1; SD = 1.45).

Regarding the utility of the editor in the field of colonoscopy, subjects reported that it could indeed be used to increase the variability in simulator training cases (mean 3.8; SD = 0.98). The editor was also rated as very useful for recreating different patient cases for visualization in classrooms for their study (mean 4.3; SD = 0.9). Additionally, creation of models for 3D printing and phantoms was suggested as an alternative usage for the editor.

**Fig. 10:**
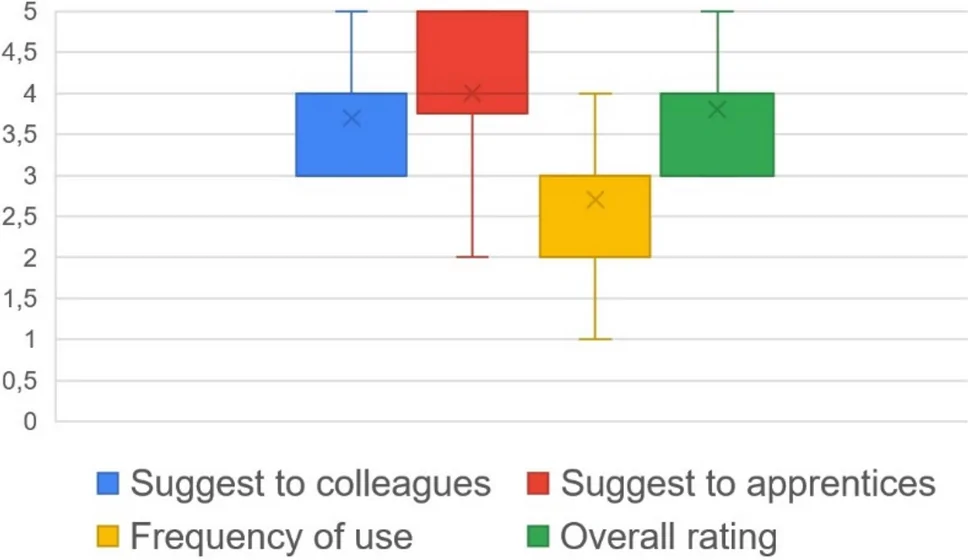
Valuation related answers boxplots

Finally, as it is shown in Fig. 10, subjects reported that they would suggest the editor to their colleagues (mean 3.7; SD = 0.64) and to colonoscopy novices (mean 4; SD = 0.89). As for an overall rating, the editor received a positive score (mean 3.8; SD = 0.6), with an average estimated frequency of use (mean 2.7; SD = 0.8).

## Discussion

From the collected data, we found that LIME was positively rated by colonoscopy practitioners. Despite having almost no experience with this type of software, LIME was rated as a user-friendly and useful tool which enables users to easily generate realistic large intestine models for colonoscopy training.

**Fig. 11:**
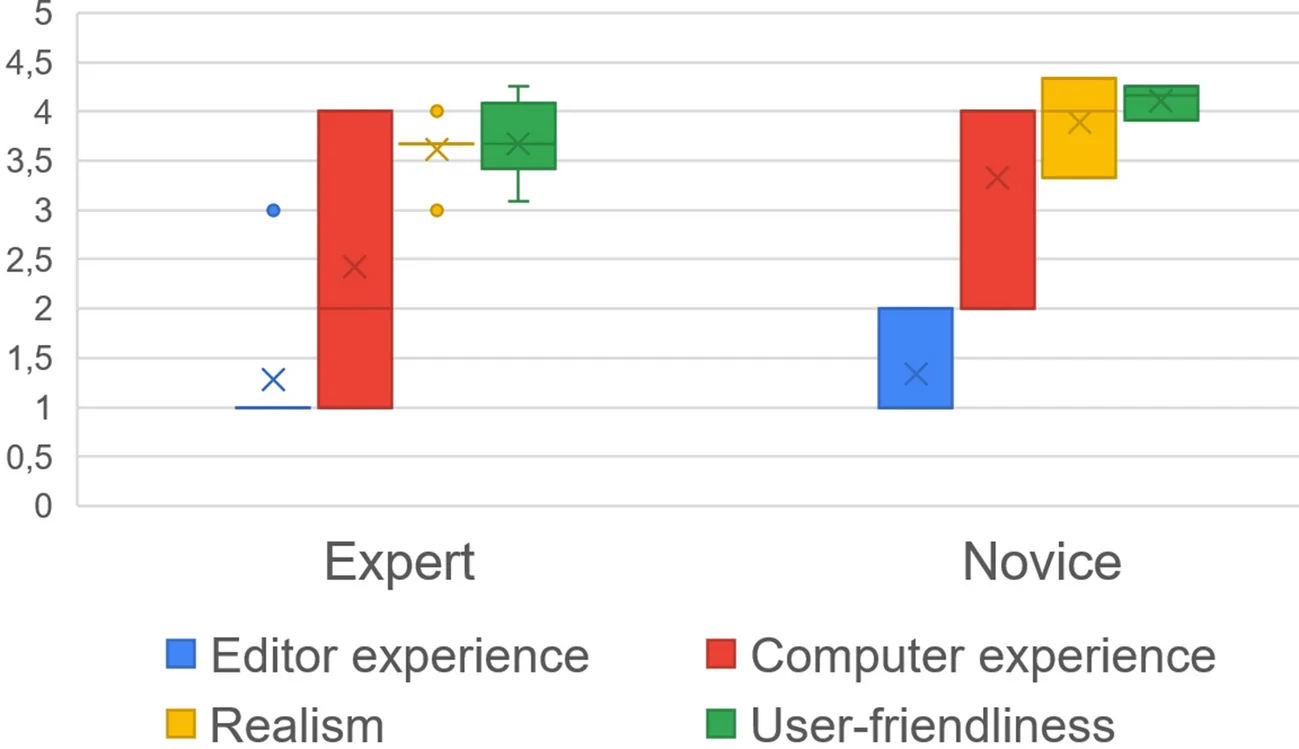
Average ratings of experts and novices regarding previous experiences, realism and user-friendliness

Regarding its realism rating, there was no relevant difference observed between novices and experts (Fig. 11). However, novices were found to rate the completeness of the anatomical details for the model significantly higher than experts. This discrepancy can be attributed to the expert eye of the seasoned practitioners, who possess a mental map of the colon refined by performing hundreds of procedures. This makes them more sensitive to specific anatomical landmarks that guide the colonoscopy navigation, such as the splenic and hepatic flexures. The fact that only experts identified these as missing elements validates their presence as a control group and highlights that while LIME outputs are positively rated, future advanced simulation scenarios require a higher degree of anatomical detail.

Despite having almost equal experience with 2D/3D editors, novices were significantly more experienced with mouse & keyboard computer games (Fig. 11). Furthermore, all novices were from the 20–35 years age group, which tends to be more familiar with new technologies and devices. This suggests that digital literacy is a primary predictor of immediate tool proficiency, as novices could adapt and transfer existing knowledge to the editor, while experts faced a steeper learning curve. This could be attributable to the observed higher overall ratings of novices in the user-friendliness and usability fields.

Another important observation is that only one expert subject (who performed over 500 colonoscopies in the last year) had any experience with computerized or virtual colonoscopy simulators. This highlights the low presence of this equipment in medical and learning institutions, where physical simulators remain as the common colonoscopy simulator, either due to the high cost or the space constraints of other simulator types.

To further contextualize the capabilities of LIME, Table 2 provides a qualitative comparison against commercial computerized and physical simulators, along with traditional modeling workflows. Since LIME is a modeling tool rather than a simulation environment, and due to the proprietary restrictions and the physical nature of some simulators, we focus on a functional comparison of the modeling pipeline capabilities instead of a quantitative comparison of underlying algorithms or mesh properties. As detailed in the table, while other solutions may excel in haptic feedback mechanisms or anatomical fidelity, LIME provides a distinct advantage in terms of model generation capabilities, availability, and independence from medical imaging data. This highlights the role of LIME as a tool to aid the generation of diverse training assets.

**Table 2:** Qualitative comparison between LIME and existing colonoscopy simulation solutions

Feature	LIME	Computerized simulators	Physical simulators	Traditional 3D modeling
Model generation	Unlimited/Parametric generation	Closed/proprietary library	Limited/Physical silicone models	Patient medical imaging data
Customizability	High (advanced control of anatomical details)	Low (scenario selection/configuration)	Low (Part-swapping/attachments)	None (static mesh derived from imaging data)
Pathology management	High (dynamic placement/projection)	Low (pre-built scenarios)	Low (pre-modeled inserts)	Medium (imaging data dependent)
Anatomical fidelity	Medium – High	High	High	Very high
Patient data required	No	No (pre-built)	No	Yes (Privacy/Cost issues)
Availability	High (Low cost/user-friendly)	Low (High cost/vendor locked modules)	Low (hardware costs)	Low (High technical skills)
Output format	JSON/BIN	Proprietary/encrypted	N/A (Physical)	Mesh (STL/OBJ/FBX)

The editor tools were rated on average as user-friendly, with higher ratings from the novices (Fig. 11). This relates to the evaluation of the proposed exercise, which was rated as simple to achieve and non-time consuming by the subjects. The tools related with pathology placement were the only ones that were rated significantly lower. Subjects positively evaluated the inside guided navigation, but they deemed the pathology projection as not user-friendly. This relates specifically to the tool implementation, where the current workflow may be imposing a high cognitive load by requiring users to simultaneously navigate in 3D space while manipulating the pathology projection. Moving along the predefined path while using the mouse to reposition the projected pathology was seen as needlessly complex by some subjects. While gaining experience from further usage could solve this, the feedback suggests that decoupling these actions into sequential steps could resolve this usability bottleneck.

**Fig. 12:**
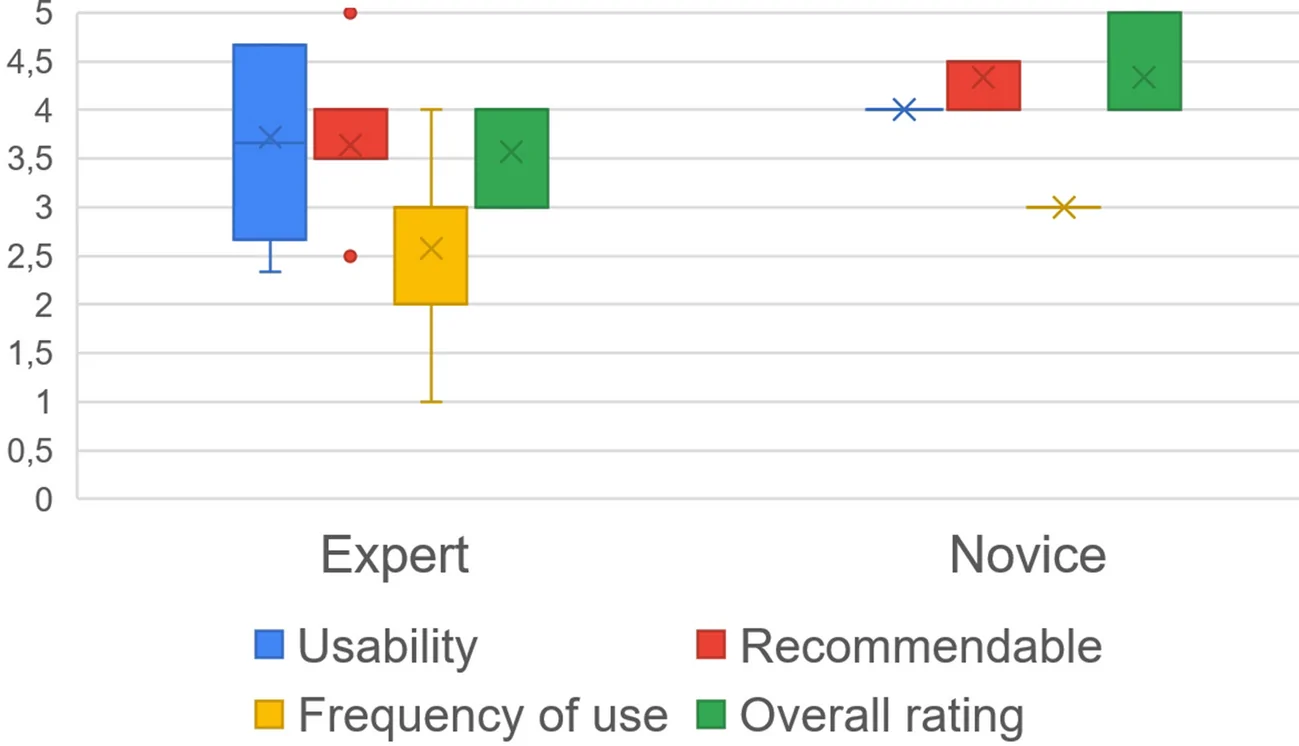
Average ratings of experts and novices for usability, probability of recommendation, frequency of use, and overall rating

Both novices and experts reported that the editor would be useful to recreate patient cases to increase training variability of simulators and for teaching purposes in classrooms (Fig. 12). In the same way, both groups indicated that they would recommend the editor to their colleagues and other novices. The most relevant difference remained in their expected frequency of use and general rating for the editor. Novices rated their expected frequency of use slightly higher than experts. This may be due to the fact that experts have surpassed their training stage, and do not really need the editor for that purpose. Instead, study of patient cases and teaching in classrooms seemed to be the main purpose suggested for the editor by the experts. Using the editor paired with a colonoscopy simulator during mainly the second to fourth year of residency was also reported as an adequate application by both experts and novices. Additionally, the editor was suggested as a tool for 3D printing and the creation of phantom models. Regarding the overall rating of the editor, as the novices rated on average every aspect of it higher than experts, the higher rating from novices was expected (Fig. 12). This feedback further highlights the value of LIME, which could serve both as an asset generator and as a tool for a skill-building sandbox.

Finally, from the pilot evaluation data, we can infer several limitations of the current LIME that will be addressed in future work:

- Anatomical details missing: Experts specifically noted splenic and hepatic flexures as missing, which novice users did not detect. This suggests that our blendshapes need to be expanded with these anatomical features and highlights the importance of expert validation in simulation design.
- Pathology placement workflow: The pathology placement tools (inside-view, projection plus mouse drag to place) was reported as cumbersome. Maintaining the guided navigation with a drag and drop feature for the pathology placement was reported several times as a preferred alternative.
- Limited sample size: Despite being enough for this pilot evaluation, the sample size (*n = *10) and the inequity between novices (*n = *3) and experts (*n = *7) distribution may have limited our capability to assess the editor in greater depth. Although these constraints potentially limit the statistical significance and generalizability of the findings, they are consistent with the exploratory nature of this pilot evaluation. For a broader future evaluation of the application, a larger and more diverse group of participants would be required to provide statistical stronger evidence of its validity.
- Validation instruments: The use of a custom questionnaire rather than standardized questionnaires or scales (e.g., SUS) limits direct comparison with other software. However, as a key objective of this pilot evaluation was to assess specific anatomical and visual fidelity along with the relevance of the editor features, domains not covered by generic approaches, a custom questionnaire based on these standards and simulation validation literature [39–48] was deemed more appropriate.

## Conclusions

In this paper, a software application for the creation and editing of large intestine models to support colonoscopy training was presented and evaluated. The proposed editor not only serves to create a broad variety of high-quality models, but also provides the means to easily edit them in a user-friendly environment without requiring specific modeling or technical knowledge. The pilot test subjects positively rated the realism of the editor, user-friendliness and usability. Subjects indicated that the editor is not only a useful tool to create large intestine models for training, but also a feasible tool for studying patient cases either in classrooms or preclinical settings. In addition, using the editor for 3D printing and phantom modeling was also suggested.

As shown in Section 2, currently available colonoscopy simulators are proprietary commercial solutions that do not allow third party contributions or innovations. Therefore, future work on our software should aim to allow the development of a community of users that can create and share large intestine models, being able to freely use and access the work of their colonoscopy colleagues.

In addition, further improvements for the editor with the suggestions gathered and the limitations found during the evaluation will be implemented. Furthermore, the editor will be integrated in a colonoscopy simulator that is currently under development. The base material of the models, textures and geometry will be improved for their usage in a physics-driven environment. Additionally, a mechanism for the automatic creation of random models will be developed, along with the integration of lightning and particle systems provided by the Unity Framework to further increase the fidelity of the models and the user experience. With these improvements, a broader validation study will be conducted to comprehensively assess the effectiveness of the application.

## Data Availability

The datasets used and/or analyzed during the current study are available from the corresponding author on reasonable request.

## Appendix 1

### Evaluation questionnaire

General questions

1. Full name
  (open response)
2. Please indicate your gender:
  - Male
  - Female
  - Other
  - Prefer not to answer
3. Please indicate your age group:
  - 20–35
  - 36–55
  - 56 or older
4. Have you previously used any of the following types of colonoscopy simulators?
  - Computerized simulators (e.g., GI Mentor, EndoSim)
  - Virtual reality simulator (e.g., GastroEx, VRCaps)
  - Both types
  - None of the above
5. If you have used any of the above simulators, please specify the product name or company.
  (open response)
6. How much experience do you have with 2D/3D editors?
  None – A lot (five-point Likert scale)
7. How much experience do you have with computer games?
  None – A lot (five-point Likert scale)
8. How many colonoscopies have you performed in the past year?
  - 0
  - 1–50
  - 51–100
  - 101–200
  - 201–500
  - More than 500
9. If you have performed none, how many have you participated in as an observer in the past year?
  - 0
  - 1–25
  - 26–50
  - More than 50
  Editor-related questions (to be answered after completing the exercises with the editor).
10. The shape of the intestinal model is realistic (excluding textures, colors…).
  Strongly disagree – Strongly agree (five-point Likert scale)
11. The anatomical details are realistic (taeniae, Houston’s valves, folds…).
  Strongly disagree – Strongly agree (five-point Likert scale)
12. All relevant anatomical details are present and I don’t miss anything significant.
  Strongly disagree – Strongly agree (five-point Likert scale)
13. If you miss any relevant anatomical details, please indicate which ones are not present.
  (open response)
14. No additional instructions are needed to use the editor during the exercises.
  Strongly disagree – Strongly agree (five-point Likert scale)
15. The user interface is intuitive.
  Strongly disagree – Strongly agree (five-point Likert scale)
16. The user interface is visually appropriate.
  Strongly disagree – Strongly agree (five-point Likert scale)
17. The editing steps are clearly defined and easy to follow.
  Strongly disagree – Strongly agree (five-point Likert scale)
18. It is easy to pan and rotate the camera view in the editor workspace.
  Strongly disagree – Strongly agree (five-point Likert scale)
19. The functionality of the editor tools is clear.
  Strongly disagree – Strongly agree (five-point Likert scale)
20. The tools for modifying the intestinal tract are easy to use.
  Strongly disagree – Strongly agree (five-point Likert scale)
21. The tools for creating intestinal-tract presets are easy to use.
  Strongly disagree – Strongly agree (five-point Likert scale)
22. The tools for modifying anatomical details are easy to use.
  Strongly disagree – Strongly agree (five-point Likert scale)
23. The tools for placing pathologies are easy to use.
  Strongly disagree – Strongly agree (five-point Likert scale)
24. The time spent completing the exercises is appropriate.
  Strongly disagree – Strongly agree (five-point Likert scale)
25. Completing the exercises is straightforward.
  Strongly disagree – Strongly agree (five-point Likert scale)
26. The provided tools would allow me to create any model of a patient or specific case.
  Strongly disagree – Strongly agree (five-point Likert scale)
27. The editor would be useful for recreating patient cases and providing greater variability for simulator training.
  Strongly disagree – Strongly agree (five-point Likert scale)
28. The editor would be useful for recreating patient cases and displaying them for classroom teaching.
  Strongly disagree – Strongly agree (five-point Likert scale)
29. Would you consider any other uses for the editor?
  (open response)
30. I would recommend the editor to my professional colleagues.
  Strongly disagree – Strongly agree (five-point Likert scale)
31. I would recommend the editor to colonoscopy trainees
  Strongly disagree – Strongly agree (five-point Likert scale)
32. At what stage of the training residency do you think the editor would be most relevant?
  (open response)
33. I would use this editor frequently.
  Strongly disagree – Strongly agree (five-point Likert scale)
34. Rate the editor.
  Very poor – Very good (five-point Likert scale)
35. Suggestions
  (open response)
